# Patient Satisfaction After Total Knee Replacement: A Systematic Review

**DOI:** 10.1007/s11420-018-9614-8

**Published:** 2018-06-05

**Authors:** Cynthia A. Kahlenberg, Benedict U. Nwachukwu, Alexander S. McLawhorn, Michael B. Cross, Charles N. Cornell, Douglas E. Padgett

**Affiliations:** Department of Orthopaedic Surgery, Hospital for Special Surgery, 535 E. 70th Street, New York, NY 10021 USA

**Keywords:** patient satisfaction, total knee arthroplasty, systematic review, PRISMA

## Abstract

**Background:**

The quality and state of satisfaction reporting after total knee replacement (TKR) is variable.

**Questions/Purposes:**

The purposes of this systematic review were (1) to examine the available literature on patient satisfaction after TKR, (2) to evaluate the quality of available evidence, and (3) to identify predictors of patient satisfaction after TKR.

**Methods:**

A systematic review of the MEDLINE database was performed. The initial search yielded 1219 studies. The inclusion criteria were English language, clinical outcome study with primary outcome related to TKR for osteoarthritis, and patient-reported satisfaction included as an outcome measure. Studies were assessed for demographics, methodology for reporting satisfaction, and factors influencing satisfaction.

**Results:**

Two hundred eight studies, including 95,560 patients who had undergone TKR, met all inclusion and exclusion criteria; 112 (53.8%) of these studies were published in the past 3 years. Satisfaction was most commonly measured using an ordinal scale. Twenty-seven studies (13%) used a validated satisfaction survey. Eighty-three percent of studies reported more than 80% satisfaction. The most commonly reported predictor of satisfaction was post-operative patient-reported functional outcome. Pre-operative anxiety/depression was the most common pre-operative predictor of dissatisfaction.

**Conclusion:**

There are numerous studies reporting patient satisfaction after TKR, and publication on the topic has been increasing over the past decade. However, the majority of studies represent lower levels of evidence and use heterogeneous methods for measuring satisfaction, and few studies use validated satisfaction instruments. In general, the majority of studies report satisfaction rates ranging from 80 to 100%, with post-operative functional outcome and relief of pain being paramount determinants for achieving satisfaction.

**Electronic supplementary material:**

The online version of this article (10.1007/s11420-018-9614-8) contains supplementary material, which is available to authorized users.

**Original Release Date: June 05, 2018**

**Expiration Date: June 05, 2020**

## Overview

The variable quality of reporting of patients’ satisfaction with total knee replacement (TKR) led the authors to conduct a systematic review. They aimed to examine the literature on patient satisfaction after TKR, to evaluate its quality, and to identify predictors of patient satisfaction with TKR. A systematic review of MEDLINE yielded 1219 studies on TKR for osteoarthritis and patient-reported satisfaction published in a ten-year period. Of these, 208 met all inclusion and exclusion criteria; 27 (13%) used a validated satisfaction survey, and 83% reported more than 80% satisfaction. Most studies represented lower levels of evidence and used heterogeneous methods for measuring satisfaction. The most common predictor of patient-reported satisfaction was post-operative functional outcome. Pre-operative anxiety or depression was the most common predictor of dissatisfaction.

## Learning Objectives

Hospital for Special Surgery CME activities are intended to improve the quality of patient care and safety. At the conclusion of the activity, the participant should be able to:

- Identify preoperative and postoperative predictors of patient satisfaction after total knee replacement.
- Discuss preoperative measures shown to improve patient satisfaction after total knee replacement and be able to implement these measures in their practice.

## Target Audience

This activity is targeted at orthopaedic surgeons. Physician assistants, residents, fellows, and medical students may also benefit from completing this activity.

## Accreditation

Hospital for Special Surgery is accredited by the Accreditation Council for Continuing Medical Education to provide continuing medical education (CME) for physicians.

## Credit Designation

Hospital for Special Surgery designates this Journal-based CME activity for a maximum of *1.0 AMA PRA Category 1 Credit(s)*™. Physicians should claim only the credit commensurate with the extent of their participation in the activity.

## Commercial Support

This journal-based activity did not receive commercial support.

## Financial Disclosure

In accordance with the Accreditation Council for Continuing Medical Education’s Standards for Commercial Support, all CME providers are required to disclose to the activity audience the relevant financial relationships of the planners, teachers, and authors involved in the development of CME content. An individual has a relevant financial relationship if he or she has a financial relationship in any amount occurring in the last 12 months with a commercial interest whose products or services are discussed in the CME activity content over which the individual has control.

It is the policy of Hospital for Special Surgery to request all financial relationships that activity directors, planning committee members, presenters, authors, and staff have with commercial interests, but to disclose to the activity audience only the relevant financial relationships.

### Activity Directors Disclosure

**Charles N. Cornell, MD**, has disclosed no relevant financial relationships.

**Joy Jacobson, MFA**, has disclosed no relevant financial relationships.

### Planning Committee Disclosure

**Charles N. Cornell, MD**, has disclosed no relevant financial relationships.

**Michael B. Cross, MD**, has disclosed no relevant financial relationships.

**Joseph D. Giordano** has disclosed no relevant financial relationships.

**Lauren Hee, MBA**, has disclosed no relevant financial relationships.

**Cynthia Ann Kahlenberg, MD**, has disclosed no relevant financial relationships.

**Alexander S. McLawhorn, MD, MBA**, has disclosed no relevant financial relationships.

**Benedict Nwachukwu, MD**, has disclosed no relevant financial relationships.

**Douglas E. Padgett, MD**, has disclosed no relevant financial relationships.

**Amy R. Stair, MS**, has disclosed no relevant financial relationships.

### Faculty Disclosure

**Charles N. Cornell, MD**, has disclosed no relevant financial relationships.

**Michael B. Cross, MD**, has disclosed no relevant financial relationships.

**Cynthia Ann Kahlenberg, MD**, has disclosed no relevant financial relationships.

**Alexander S. McLawhorn, MD, MBA**, has disclosed no relevant financial relationships.

**Benedict Nwachukwu, MD**, has disclosed no relevant financial relationships.

**Douglas E. Padgett, MD**, has disclosed no relevant financial relationships.

### OCME/CME Committee Disclosure

Hospital for Special Surgery Office of CME staff and CME committee members have no relevant financial relationships to disclose regarding this activity.

### Authors/Faculty

**Charles N. Cornell, MD**

Editor, HSS Journal

Attending Orthopaedic Surgeon

Hospital for Special Surgery

Professor of Clinical Orthopaedic Surgery

Weill Cornell Medical College

**Michael B. Cross, MD**

Assistant Attending Orthopaedic Surgeon

Hospital for Special Surgery

Assistant Professor of Orthopaedic Surgery

Weill Cornell Medical College

**Cynthia Ann Kahlenberg, MD**

Orthopaedic Resident

PGY 3

Hospital for Special Surgery

**Alexander S. McLawhorn, MD, MBA**

Assistant Attending Orthopaedic Surgeon

Hospital for Special Surgery

Instructor of Orthopaedic Surgery

Weill Cornell Medical College

**Benedict Nwachukwu, MD**

Orthopaedic Resident

PGY 5

Hospital for Special Surgery

**Douglas E. Padgett, MD**

Chief, Adult Reconstruction and Joint Replacement Service

Associate Attending Orthopaedic Surgeon

Hospital for Special Surgery

Associate Professor of Orthopaedic Surgery

Weill Cornell Medical College

**Instructions for Post-test, Course Evaluation and CME Credit**:

In order to earn CME credit, you must complete an online post-test and evaluation following the completion of this activity. There is a passing requirement of 100%. Once you complete the post-test and subsequent evaluation, a certificate will be available for you to print.

For questions related to the post-test and subsequent evaluation, please contact HSS Journal at jacobsonj@hss.edu or 646-797-8509.

*To Complete the Activity Online*

1. Go to the HSS Journal homepage at www.springer.com/hss.
2. Click on “CME and Free-to-Access Articles” tab.
3. Click on “Patient Satisfaction After Total Knee Replacement” to view the full-text pdf article.
4. After you have read the article click on “Complete the Current CME Test Online” to complete the test.
5. Once you have passed the post-test with a score of 100%, you will be able to complete the evaluation. You will then be able to print your CME certificate.

## Introduction

Total knee replacement (TKR) is one of the most common orthopaedic surgeries, with over 700,000 procedures performed in 2014 in the USA and projected increases in the coming decades [6, 13]. TKR aims to reduce pain, restore function, and improve the quality of life for patients with end-stage knee arthritis [2]. In this regard, TKR is a clinically proven and cost-effective procedure [17]. However, a significant number of patients are not satisfied with the results of TKR [3].

Orthopaedic thought leaders have called for improvements in satisfaction reporting as a way of demonstrating the value of orthopedic procedures [5]. Prior reports of satisfaction in orthopaedic literature have demonstrated low methodological quality, with no uniform standard for determining patient satisfaction [9–11]. In the USA, government initiatives through the Centers for Medicare and Medicaid Services (CMS) and the Affordable Care Act have emphasized the importance of patient satisfaction and have suggested linking patient satisfaction reporting to reimbursement [5]. With increasing attention on healthcare reform and a national focus on cost containment, patient satisfaction has been proposed as an outcome measure to define the quality and value of elective procedures. TKR is the most commonly performed orthopaedic procedure, yet the existing evidence base for patient satisfaction after TKR has not been analyzed thoroughly.

The purpose of this systematic review was to examine the available literature on patient satisfaction after TKR and to evaluate the quality of available evidence. The second purpose was to identify predictors of patient satisfaction after TKR. We anticipated a large number of studies, given the increased interest in patient satisfaction in recent years, but we hypothesized that the quality of satisfaction reporting in the TKR literature would be low, with no uniform method of evaluating patient satisfaction after TKR.

## Materials and Methods

A systematic review of the MEDLINE database using the PubMed interface was performed in February 2017. The results were limited to articles published between January 1, 2007, and January 1, 2017. The search terms used were “knee replacement satisfaction” and “knee arthroplasty satisfaction.” The Preferred Reporting Items for Systematic Reviews and Meta-Analyses (PRISMA) guidelines and checklist were employed [16]. The study protocol was registered with PROSPERO (CRD42017068659).

**Fig. 1:**
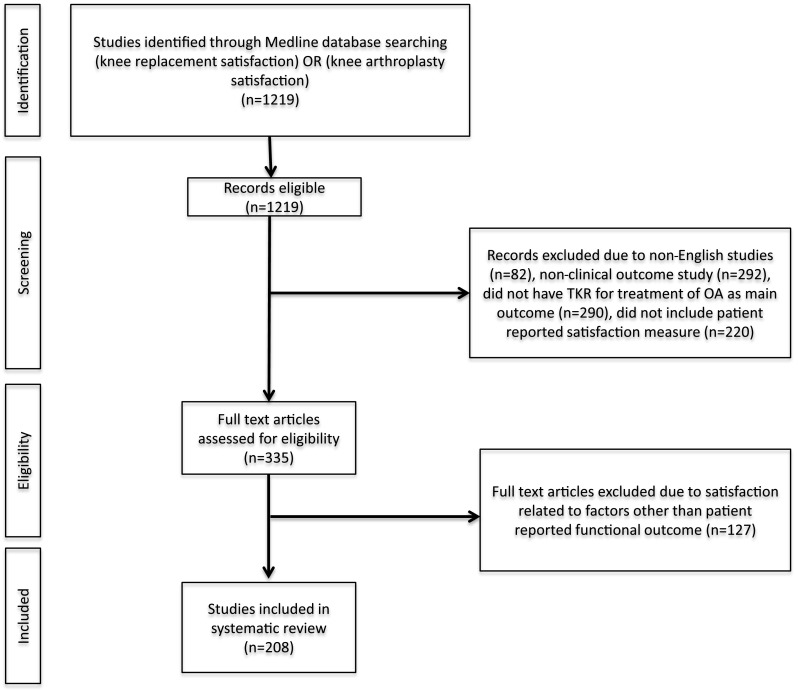
Preferred Reporting Items for Systematic Reviews and Meta-Analyses (PRISMA) flow chart illustration of study inclusion and exclusion criteria

The initial search yielded 1219 articles. Each article’s abstract was reviewed for the inclusion criteria, which were English language, clinical outcome study with primary outcome related to TKR for osteoarthritis, and patient-reported satisfaction included as an outcome measure (Fig. 1). The satisfaction outcome measure was limited to satisfaction with clinical outcome of TKR. Thus, studies focusing on other areas of satisfaction such as anesthesia or physical therapy were excluded (*n* = 127). Studies that combined outcomes of unicondylar knee replacement and TKR, hip replacement and TKR, and primary TKR with revision TKR were included if the studies reported clinical outcomes of each type of surgery separately but excluded if only aggregate outcomes were reported.

The following data points were extracted from each study: region of origin, level of evidence, number and gender of patients, method of reporting satisfaction and whether or not it was validated in prior literature, percentage of patients who were overall satisfied after TKR, other outcome measures used, time intervals when satisfaction was measured, reporting of pre-operative expectations or pre-operative satisfaction, primary variable under investigation in each study, and predictors of satisfaction or dissatisfaction. Furthermore, the quality of each study was evaluated using the Methodological Index for Non-Randomized Studies (MINORS) criteria [19] or the Jadad score [7] for randomized controlled trials. The MINORS criteria include a 24-point scale for comparative studies and a 16-point scale for non-comparative studies. The Jadad Score is a 5-point scale intended for evaluation of the quality of randomized studies. Higher scores on both scales indicate better study quality.

The heterogeneity of included studies and reported outcome data precluded a formal meta-analysis. Thus, statistics were primarily descriptive and each study was analyzed qualitatively.

## Results

**Fig. 2:**
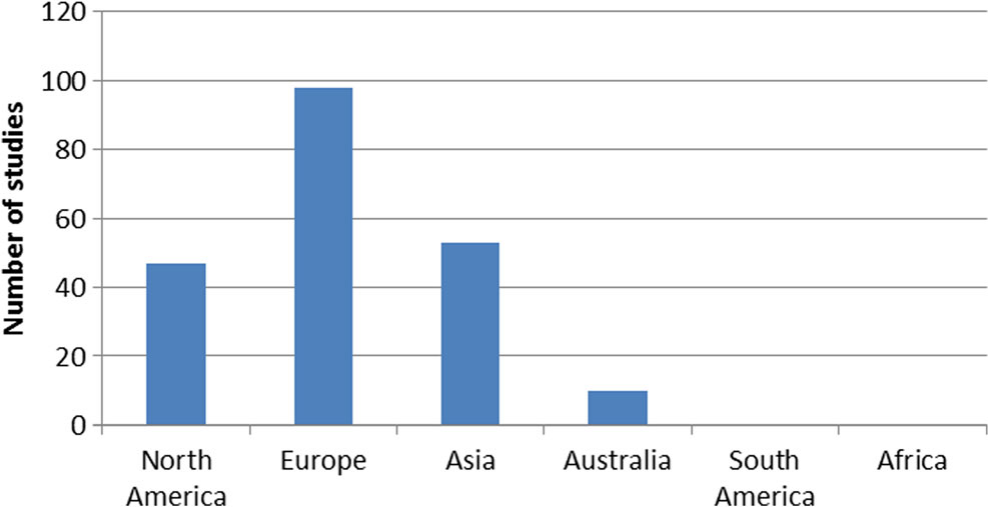
Number of publications by region

**Fig. 3:**
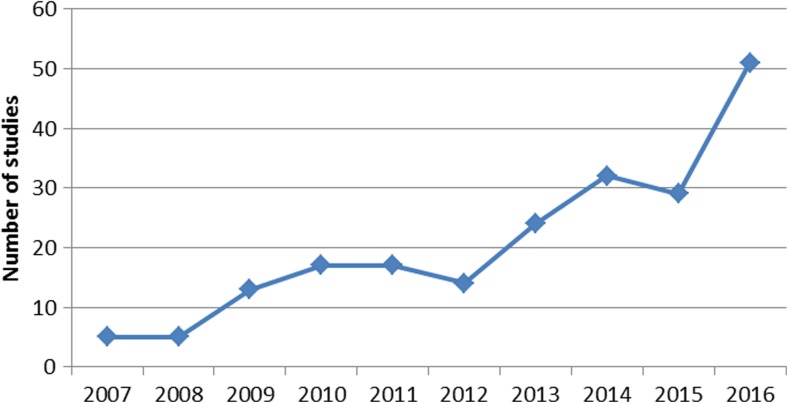
Trend in publication number over time

Of the 1219 studies identified in the initial search, 208 studies (17.1%) met all inclusion and exclusion criteria (see Online Resource 1 for a list of references). These studies included a total of 95,560 patients who had undergone TKR. Thirty-seven studies (17.7%) were randomized controlled trials. The mean Jadad Score was 3.51 (range, 0 to 5). The remaining studies were evaluated with the MINORS criteria and had a mean score of 11.0 (range, 5 to 20). The highest number of studies were level-IV evidence, and the most common journal was *Journal of Arthroplasty* (Table 1). Included studies came from a total of 48 different journals. The region producing the highest number of studies was Europe (Fig. 2). The number of publications per year reporting satisfaction and meeting inclusion criteria rose from five publications in the first year under study to 51 publications in the most recent year under study (Fig. 3).

**Table 1:** Characteristics of included studies

	*N* (208)	%
Level of evidence		
Level I	29	13.9
Level II	51	24.5
Level III	50	24.0
Level IV	78	37.5
Most common journals		
*Journal of Arthroplasty*	50	24.0
*Knee Surgery, Sports Traumatology, Arthroscopy*	17	8.2
*Journal of Bone and Joint Surgery British*	15	7.2
*Bone and Joint Journal (*formerly *JBJS Br)*	13	6.3
*The Knee*	12	5.8
*Clinical Orthopaedics and Related Research*	10	4.8
*Archives of Orthopaedic and Trauma Surgery*	8	3.8
*International Orthopaedics*	8	3.8
*Journal of Bone and Joint Surgery American*	8	3.8
*Acta Orthopaedica*	7	3.4
*BMC Musculoskeletal Disorders*	6	2.9
*Journal of Knee Surgery*	4	1.9
*Orthopedics*	4	1.9
*PLoS One*	4	1.9

The method of evaluating patient satisfaction was clearly defined in the methods section of 164 (78.8%) of the 208 studies (Table 2). The most commonly used method of satisfaction reporting was with a single question about overall satisfaction that could be answered on an ordinal scale (e.g., very satisfied, somewhat satisfied, dissatisfied, very dissatisfied). Sixteen studies (7.7%) used multiple methods to define satisfaction, most commonly a combination of an ordinal scale plus a numeric scale or a binary question asking whether the patient would undergo surgery again. Fifty-three studies (25.5%) asked in one questionnaire about satisfaction in multiple domains, most commonly pain, function, and overall outcome.

**Table 2:** Reporting of satisfaction

	*N* (208)	%
Methodology for assessing satisfaction described	164	78.8
Used a validated method of satisfaction	27	13.0
Method for assessing satisfaction^a^		
Numeric or VAS-type 0–10 scale	35	16.8
Numeric or VAS-type 0–100 scale	20	9.6
Binary scale (satisfied: yes/no)	21	10.1
Ordinal scale (e.g., very satisfied, satisfied, neutral, dissatisfied)	127	61.1
Willingness to undergo surgery again	13	6.3
Would recommend surgery	5	2.4
Primary variable under investigation		
General outcome	88	42.3
Implant	46	22.1
Surgical technique	35	16.8
Other	32	15.4
Anatomic alignment	7	3.4
Satisfaction measured at multiple time intervals	31	14.9
Pre-operative satisfaction measured	8	3.8
Pre-operative expectations measured	17	8.2
Differentiation between satisfaction with outcome and process of care	3	1.4

^a^Satisfaction totals > 100% because some studies used multiple methods for assessing satisfaction

*VAS* visual analogue scale

Among the 27 studies (13%) using a previously validated method of satisfaction reporting, 15 (55.6%) used the 2011 Knee Society Knee Scoring System [18], which evaluates patient satisfaction in the domains of pain while sitting, pain while lying in bed, function while getting out of bed, function while performing light household duties, and function while performing recreational activities. Six (22.2%) of the 27 studies used a four-item questionnaire in which each item was scored on a Likert scale. This questionnaire, which was validated by Mahomed et al. for primary hip and knee arthroplasty [14], evaluates overall satisfaction, satisfaction with pain relief, satisfaction with ability to do housework, and satisfaction with ability to perform recreational activities.

**Fig. 4:**
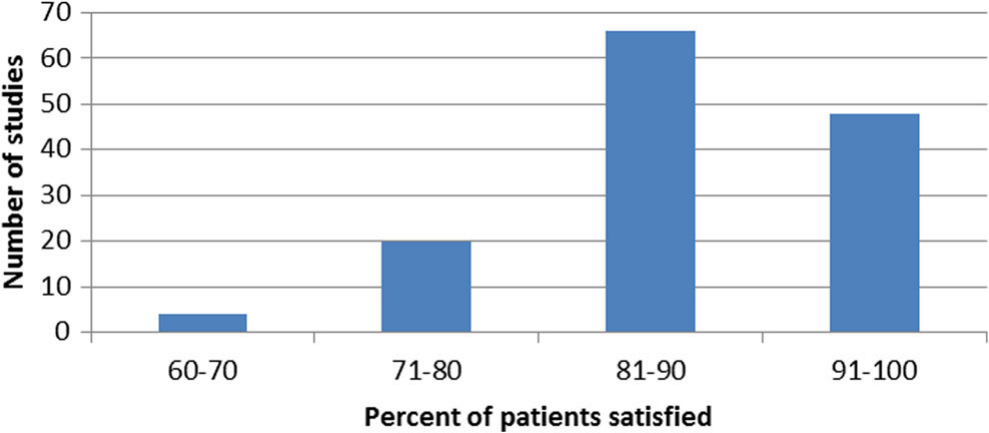
Percentage of patients classified as “satisfied” by each individual study; in general, most studies considered patients “satisfied” if they scored 4 or 5 (satisfied or very satisfied) on a 5-point ordinal scale or ≥ 7 on a 10-point scale

The percentage of patients who were satisfied overall was reported by 138 studies (66.3%), while the remainder did not report an overall percentage of satisfied patients. In the studies that did report this, the percentage of satisfied patients reported ranged from 65 to 100%, with the majority of studies (82.6%) reporting greater than 80% satisfaction (Fig. 4). The median reported percentage of satisfied patients was 88.9%. Among studies that reported a numeric 0–10 scale for satisfaction, the range of means reported was 7.0 to 10.0. Most studies only measured satisfaction at the final follow-up point, but 31 studies (14.9%) reported satisfaction at multiple time points. The mean time of reporting satisfaction was 38.2 months post-operatively (range, 0.33 to 199 months). Eight studies (3.8%) measured pre-operative satisfaction and 17 studies (8.2%) measured pre-operative expectations.

Among the 29 level-I studies included in the review, 15 (51.7%) used a numeric 0–10 or 0–100 scale to evaluate satisfaction. Eight (27.6%) used an ordinal scale. Only three (10.3%) of the 29 studies evaluated satisfaction across multiple domains or with multiple questions. None measured pre-operative satisfaction or expectations. Seventeen (58.6%) of the level-I studies evaluated implant design as the main outcome variable, and twelve (41.4%) evaluated surgical technique.

The included studies reported a multitude of pre-operative, intra-operative, and post-operative predictors of satisfaction and dissatisfaction after TKR (Table 3). The most commonly reported predictor of satisfaction was higher absolute post-operative patient-reported functional score. This was followed by less persistent pain and a higher improvement from pre-operative to post-operative function. Seven studies reported fulfillment of expectations as a predictor of post-operative satisfaction. Pre-operative anxiety and/or depression was the most common pre-operative predictor of dissatisfaction, and persistent pain was the most common post-operative predictor.

**Table 3:** Predictors of satisfaction and dissatisfaction listed by more than one study

Predictors of satisfaction	Predictors of dissatisfaction
Preoperative factors Higher-grade osteoarthritis (4) Higher baseline patient reported function (3) Better emotional/mental health (2) Older age (3) Male gender (2) Implants Triathlon knee (compared to Kinemax) (2) Rotating platform (compared to medial pivot fixed bearing) (4) Postoperative factors Less pain (12) No complication (2) Fulfillment of expectations (7) More improvement on functional score (8) Higher postoperative patient-reported function (16) Better function on walk test (2) Less knee stiffness/improvement in ROM (5) Higher general health score (2)	Preoperative factors Anxiety/depression/poorer mental health (10) Lack of social support (2) Younger patient age (2) Less-severe arthritis (3) Female gender (2) Lower income (2) Post-operative Factors Longer hospital stay (2) Flexion contracture/diminished ROM (3) Persistent pain (9) Worse patient-reported function (3) Requiring readmission or revision surgery (3)

Number in parentheses indicates number of studies reporting that predictor

*ROM* range of motion

## Discussion

As anticipated, this systematic review of the total knee arthroplasty literature revealed a large number of studies reporting patient satisfaction, with a steady increase in the number of studies over the past decade. This likely reflects the growing interest in satisfaction as an important outcome measure following TKR, as it has become closely linked with patient-focused outcomes, physician evaluation, and value-based initiatives [5].

The quality of the studies reporting patient satisfaction was moderately low, with the majority reporting levels III or IV evidence and a mean MINORS score of 9.1 for non-comparative studies and 15.1 for comparative studies. Furthermore, the methodology of reporting patient satisfaction was highly variable. Most studies did not use validated methods of reporting satisfaction and only reported satisfaction in one domain. Of the studies that did use a validated method of reporting satisfaction, more than half employed scores from the 2011 Knee Society Knee Scoring System [18], which is a comprehensive validated patient- and surgeon-reported outcome measure that includes patient reports of both expectations and satisfaction. It asks about satisfaction across multiple domains and is a highly useful instrument for comprehensively evaluating patients before and after TKR. A small number of studies used the Self-Administered Patient Satisfaction Scale [14], which is a brief four-question survey about satisfaction in multiple domains. Future efforts should be placed on unifying the method of assessing patient satisfaction so that it can be compared as an outcome measure across different studies.

This review identified a multitude of predictors of satisfaction and dissatisfaction reported in the literature. The most commonly reported predictors of satisfaction were higher overall post-operative function, greater improvement in function from pre-operative to post-operative levels, and decreased pain. Fulfillment of expectations was also reported as a key predictor of satisfaction after TKR. Bourne et al. reported in a study of 1703 patients undergoing primary TKR that expectations not fulfilled 1 year post-operatively were associated with a 10.7 times higher risk of dissatisfaction compared to expectations fulfilled [3]. However, only 8.2% of the studies in this review evaluated pre-operative expectations. Given the magnitude of the association between patient expectations and patient satisfaction, pre-operative collection of patient expectation data may be critical to optimizing patient satisfaction. Evaluating pre-operative expectations can allow for a structured discussion between patient and surgeon, in which patients can be counseled about their anticipated outcomes, vis-à-vis shared decision making [15]. Setting appropriate expectations is likely a crucial step in optimizing patient satisfaction after TKR.

The majority of studies reported more than 80% patient satisfaction, while a small number reported overall satisfaction in the 60-to-80% range. Therefore, although the reported satisfaction is generally high, around one in five patients may be dissatisfied with their elective TKR. Many studies reported factors that may place patients at higher risk for dissatisfaction. The most frequently reported predictors of dissatisfaction included persistent pain after surgery and anxiety, depression, or poorer mental health as measured by clinical diagnosis or pre-operative questionnaires. Persistent pain after TKR has been a subject of ongoing study for many decades and continues to be a problem for some patients. In studies that reported anxiety, depression, and poorer mental health as predictors of dissatisfaction, the recommendation was not to exclude these patients from surgery, but rather to identify mental health issues pre-operatively and address them or use this information in counseling patients about the outcomes of TKR [1, 4]. Evaluating predictors of dissatisfaction can be helpful in targeting at-risk patients and providing pre-operative counseling to set appropriate expectations for TKR.

A recent review of hip arthroplasty satisfaction reporting limited to US literature found a similar problem with variable methods of reporting patient satisfaction [11]. In the hip literature, the most commonly used reporting method was a numeric 0–10 scale for overall satisfaction. Predictors of better patient satisfaction were similarly decreased pain, along with leg length equality and hip stability. Also similar to TKR, hip arthroplasty patients with lower scores on mental health questionnaires had poorer satisfaction post-operatively. The hip literature included 24 studies over a 10-year period reporting satisfaction in all types of domains, including anesthesia, pain control, post-operative physical therapy, and functional outcome. In the present review, the TKR literature in North America included 47 studies reporting on satisfaction specifically with functional outcome. Overall, it appears that the TKR satisfaction literature is more robust than that of hip replacement.

Our review is not without limitations. First, it included a heterogeneous patient population and studies with heterogeneous methodologies. Thus, we were unable to perform a detailed meta-analysis of the data. Furthermore, our study included papers with study populations from four different continents. Prior reports have suggested that cultural differences and variations in healthcare systems may contribute to patient-reported satisfaction [8, 12]. Therefore, the satisfaction findings from the included studies may not be comparable. However, given that our aim was to evaluate patient satisfaction reporting across the TKR literature, we determined that it would be most useful to evaluate the entire body of available evidence in the selected time period, rather than to exclude studies based on geography. Finally, the review was limited by the quality of the available studies. The highest number of studies were level-IV evidence. Future higher-level studies are needed to assess the optimal methods for defining patient satisfaction after TKR.

In conclusion, numerous studies report satisfaction after TKR, and publication on the topic has been increasing over the past decade. However, the majority of studies represent lower levels of evidence and use variable methods for measuring and reporting satisfaction, and few studies use validated satisfaction instruments. In general, the majority of studies report satisfaction rates ranging from 80 to 100%, with post-operative functional outcome and relief of pain being paramount determinants of satisfaction. Future research should focus on standardizing patient satisfaction reporting and defining ways to optimize patient satisfaction after TKR. Specialty societies may play a leading role in promoting the use of the available validated satisfaction instruments. Institutional registries and clinical follow-up studies should include validated satisfaction tools in order to define ways of improving patient satisfaction after TKR and to determine what techniques lead to the highest levels of satisfaction. Consensus regarding a validated, patient-centered instrument to measure satisfaction after TKR would improve the quality of satisfaction reporting and facilitate comparisons across studies.

## Electronic supplementary material

ESM 1 (PDF 1.19 mb)

ESM 2 (PDF 447 kb)
