# Supplementary material for: Patient Satisfaction After Total Knee Replacement: A Systematic Review
Source: HSS J. 2018 Jun 5;14(2):192–201. doi: 10.1007/s11420-018-9614-8 (PMC6031540; doi:10.1007/s11420-018-9614-8)
Supplement: Supplementary file 2 — (PDF 447 kb) [file 11420_2018_9614_MOESM2_ESM.pdf]

Studies for: Patient Satisfaction After Total Knee Replacement: A Systematic Review

Title	Author	Journal	Year	PMID
Total knee arthroplasty with an oxidised zirconium femoral component: ten-year survivorship analysis.	Ahmed I, Salmon LJ, Waller A, Watanabe H, Roe JP, Pinczewski LA.	Bone Joint J	2016	26733516
Total Knee Arthroplasty due to Knee Osteoarthritis: Risk Factors for Persistent Postsurgical Pain.	Albayrak I, Apiliogullari S, Erkocak OF, Kavalci H, Ozerbil OM, Levendoglu F.	J Natl Med Assoc	2016	27979009
Similar patient-reported outcomes and performance after total knee arthroplasty with or without patellar resurfacing.	Ali A, Lindstrand A, Nilsdotter A, Sundberg M.	Acta Orthop	2016	27212102
Preoperative Anxiety and Depression Correlate With Dissatisfaction After Total Knee Arthroplasty: A Prospective Longitudinal Cohort Study of 186 Patients, With 4-Year Follow-Up.	Ali A, Lindstrand A, Sundberg M, Flivik G.	J Arthroplasty	2016	27692782
The Quality of Life (QOL) after Total Knee Arthroplasties among Saudi Arabians: A Pilot Study.	Al-Omran AS.	Int J Biomed Sci	2014	25324701
Intraoperative ligament laxity influences functional outcome 1 year after total knee arthroplasty.	Aunan E, Kibsgård TJ, Diep LM, Röhrl SM.	Knee Surg Sports Traumatol Arthrosc	2015	24917538
Patellar resurfacing in total knee arthroplasty: functional outcome differs with different outcome scores: A randomized, double-blind study of 129 knees with 3 years of follow-up.	Aunan E, Næss G, Clarke-Jenssen J, Sandvik L, Kibsgård TJ.	Acta Orthop	2016	26540368
Mid-term survival following primary hinged total knee replacement is good irrespective of the indication for surgery.	Baker P, Critchley R, Gray A, Jameson S, Gregg P, Port A, Deehan D.	Knee Surg Sports Traumatol Arthrosc	2014	23238923
Influence of body mass index (BMI) on functional improvements at 3 years following total knee replacement: a retrospective cohort study.	Baker P, Muthumayandi K, Gerrand C, Kleim B, Bettinson K, Deehan D.	PLoS One	2013	23527090

Patient satisfaction with total knee replacement cannot be predicted from pre-operative variables alone: A cohort study from the National Joint Registry for England and Wales.	Baker PN, Rushton S, Jameson SS, Reed M, Gregg P, Deehan DJ.	Bone Joint J	2013	24078532
The role of pain and function in determining patient satisfaction after total knee replacement. Data from the National Joint Registry for England and Wales.	Baker PN, van der Meulen JH, Lewsey J, Gregg PJ; National Joint Registry for England and Wales..	J Bone Joint Surg Br	2007	17673581
Impact of socioeconomic factors on outcome of total knee arthroplasty.	Barrack RL, Ruh EL, Chen J, Lombardi AV Jr, Berend KR, Parvizi J, Della Valle CJ, Hamilton	Clin Orthop Relat Res	2014	23633182
Total knee arthroplasty in patients with fibromyalgia.	Bican O, Jacovides C, Pulido L, Saunders C, Parvizi J.	J Knee Surg	2011	22303755
Influence of somatization and depressive symptoms on the course of pain within the first year after uncomplicated total knee replacement: a prospective study.	Bierke S, Häner M, Petersen W.	Int Orthop	2016	26820743
A simple patient-driven tool to measure the results of total knee arthroplasty.	Bindelglass DF, Marchetti DJ, Myers C.	Conn Med	2010	20218047
Electromagnetic navigation in total knee arthroplasty-a single center, randomized, single-blind study comparing the results with conventional techniques.	Blyth MJ, Smith JR, Anthony IC, Strict NE, Rowe PJ, Jones BG.	J Arthroplasty	2015	25263246
Range of motion and patient satisfaction with traditional and high-flexion rotating-platform knees.	Boese CK, Gallo TJ, Plantikow CJ.	Iowa Orthop J	2011	22096424
Knee Kinematic and Clinical Outcomes Evolution Before, 3 Months, and 1 Year After Total Knee Arthroplasty.	Bonnefoy-Mazure A, Armand S, Sagawa Y Jr, Suvà D, Miozzari H, Turcot K.	J Arthroplasty	2016	28007371
Can patients really do sport after TKA?	Bonnin M, Laurent JR, Parratte S, Zadegan F, Badet R, Bissery A.	Knee Surg Sports Traumatol Arthrosc	2010	20033676
Comparing patient outcomes after THA and TKA: is there a difference?	Bourne RB, Chesworth B, Davis A, Mahomed N, Charron K.	Clin Orthop Relat Res	2010	19760472

Patient satisfaction after total knee arthroplasty: who is satisfied and who is not?	Bourne RB, Chesworth BM, Davis AM, Mahomed NN, Charron KD.	Clin Orthop Relat Res	2010	19844772
Midterm results using a medial pivot total knee replacement compared with the Australian National Joint Replacement Registry data.	Brinkman JM, Bubra PS, Walker P, Walsh WR, Bruce WJ.	ANZ J Surg	2014	24165225
Surgeon satisfaction agreement after total knee arthroplasty using a visual analogue scale: a single surgeon series.	Brokelman RB, van Loon CJ, Boog GJ.	Arch Orthop Trauma Surg	2008	17187261
A prospective randomized clinical trial of patellar resurfacing and nonresurfacing in bilateral TKA.	Burnett RS, Boone JL, McCarthy KP, Rosenzweig S, Barrack RL.	Clin Orthop Relat Res	2007	17589364
Patellar resurfacing compared with nonresurfacing in total knee arthroplasty. A concise follow-up of a randomized trial.	Burnett RS, Boone JL, Rosenzweig SD, Steger-May K, Barrack RL.	J Bone Joint Surg Am	2009	19884428
Outcomes of 447 SCORE® highly congruent mobile-bearing total knee arthroplasties after 5-10 years follow-up.	Châtain F, Gaillard TH, Denjean S, Tayot O.	Orthop Traumatol Surg Res	2013	23988419
Activity levels and participation in physical activities by Korean patients following total knee arthroplasty.	Chang MJ, Kim SH, Kang YG, Chang CB, Kim TK.	BMC Musculoskelet Disord	2014	25030804
Are Midterm Patient-Reported Outcome Measures Between Rotating-Platform Mobile-Bearing Prosthesis and Medial-Pivot Prosthesis Different? A Minimum of 5-Year Follow-Up Study.	Choi NY, In Y, Bae JH, Do JH, Chung SJ, Koh IJ.	J Arthroplasty	2016	27667531
Comparison between standard and high-flexion posterior-stabilized rotating-platform mobile-bearing total knee arthroplasties: a randomized controlled study.	Choi WC, Lee S, Seong SC, Jung JH, Lee MC.	J Bone Joint Surg Am	2010	20952606

Patient satisfaction after total knee arthroplasty is affected by their general physical well-being.	Clement ND, Burnett R.	Knee Surg Sports Traumatol Arthrosc	2013	23670127
Socioeconomic status affects the Oxford knee score and short-form 12 score following total knee replacement.	Clement ND, Jenkins PJ, MacDonald D, Nie YX, Patton JT, Breusch SJ, Howie CR, Biant LC.	Bone Joint J	2013	23307673
Diabetes does not influence the early outcome of total knee replacement: a prospective study assessing the Oxford knee score, short form 12, and patient satisfaction.	Clement ND, MacDonald D, Burnett R, Breusch SJ.	Knee	2013	23993274
Predicting patient satisfaction using the Oxford knee score: where do we draw the line?	Clement ND, Macdonald D, Burnett R.	Arch Orthop Trauma Surg	2013	23525559
Primary total knee replacement in patients with mental disability improves their mental health and knee function: a prospective study.	Clement ND, MacDonald D, Burnett R.	Bone Joint J	2013	23450021
The outcome of primary total hip and knee arthroplasty in patients aged 80 years or more.	Clement ND, MacDonald D, Howie CR, Biant LC.	J Bone Joint Surg Br	2011	21911540
Post-operative Oxford knee score can be used to indicate whether patient expectations have been achieved after primary total knee arthroplasty.	Clement ND, MacDonald D, Patton JT, Burnett R.	Knee Surg Sports Traumatol Arthrosc	2015	24488224
Total knee replacement in patients with concomitant back pain results in a worse functional outcome and a lower rate of satisfaction.	Clement ND, MacDonald D, Simpson AH, Burnett R.	Bone Joint J	2013	24293592
Better functional outcome after single-radius TKA compared with multi-radius TKA.	Collados-Maestre I, Lizaur-Utrilla A, Gonzalez-Navarro B, Miralles-Muñoz FA, Marco-Gomez L, Lopez-Prats FA, Gil-Guillen V.	Knee Surg Sports Traumatol Arthrosc	2016	27522590

Concomitant low back pain impairs outcomes after primary total knee arthroplasty in patients over 65Â years: a prospective, matched cohort study.	Collados-Maestre I, Lizaur-Utrilla A, Martinez-Mendez D, Marco-Gomez L, Lopez-Prats FA.	Arch Orthop Trauma Surg	2016	27699468
Patient-reported quality of life after primary major joint arthroplasty: a prospective comparison of hip and knee arthroplasty.	Dailiana ZH, Papakostidou I, Varitimidis S, Liaropoulos L, Zintzaras E, Karachalios T, Michelinakis E, Malizos KN.	BMC Musculoskelet Disord	2015	26612135
Does BMI influence clinical outcomes after total knee arthroplasty?	Daniilidis K, Yao D, Gosheger G, Berssen C, Budny T, Dieckmann R, Höll S.	Technol Health Care	2016	26757443
Primary total knee arthroplasty in patients with fibromyalgia.	D'Apuzzo MR, Cabanela ME, Trousdale RT, Sierra RJ.	Orthopedics	2012	22310402
Total knee arthroplasty of the stiff knee: three hundred and four cases.	Debette C, Lustig S, Servien E, Lording T, Villa V, Demey G, Neyret P.	Int Orthop	2014	24363046
Does greater knee flexion increase patient function and satisfaction after total knee arthroplasty?	Devers BN, Conditt MA, Jamieson ML, Driscoll MD, Noble PC, Parsley BS.	J Arthroplasty	2011	20413247
Comparison of patient reported outcomes after Triathlon® and Kinemax Plus prostheses.	Dixon S, Blom AW, Whitehouse MR, Wylde V.	Ann R Coll Surg Engl	2014	24417833
Patient satisfaction after posterior-stabilized total knee arthroplasty: a functional specific analysis.	Du H, Tang H, Gu JM, Zhou YX.	Knee	2014	24835581
Anxiety and depressive symptoms before and after total hip and knee arthroplasty: a prospective multicentre study.	Duivenvoorden T, Vissers MM, Verhaar JA, Busschbach JJ, Gosens T, Bloem RM, Bierma-Zeinstra SM, Reijman M.	Osteoarthritis Cartilage	2013	24012622
Patelloplasty with patellar decompression to relieve anterior knee pain in total knee arthroplasty.	Ertürk C, Altay MA, Işikan UE.	Acta Orthop Traumatol Turc	2011	22245819
Mid-term outcomes of primary constrained condylar knee arthroplasty for severe knee deformity.	Feng XB, Yang C, Fu DH, Ye SN, Liu XZ, Chen Z, Rai S, Yang SH.	J Huazhong Univ Sci Technolog Med Sci	2016	27072968

To Cement or Not? Two-Year Results of a Prospective, Randomized Study Comparing Cemented Vs. Cementless Total Knee Arthroplasty (TKA).	Fricka KB, Sritulanondha S, McAsey CJ.	J Arthroplasty	2015	26118567
Quadriceps strength affects patient satisfaction after total knee arthroplasty.	Furu M, Ito H, Nishikawa T, Nankaku M, Kuriyama S, Ishikawa M, Nakamura S, Azukizawa M, Hamamoto Y, Matsuda S.	J Orthop Sci	2016	26755384
Change of impairment, disability and patient satisfaction after total knee arthroplasty in secondary care practice.	Genêt F, Schnitzler A, Lapeyre E, Roche N, Autret K, Fermanian C, Poiraudeau S.	Ann Readapt Med Phys	2008	18801590
The Impact of Personality Traits on the Outcome of Total Knee Arthroplasty.	Giurea A, Fraberger G, Kolbitsch P, Lass R, Schneider E, Kubista B, Windhager R.	Biomed Res Int	2016	26989686
Patient-Reported Outcomes, Quality of Life, and Satisfaction Rates in Young Patients Aged 50 Years or Younger After Total Knee Arthroplasty.	Goh GS, Liow MH, Bin Abd Razak HR, Tay DK, Lo NN, Yeo SJ.	J Arthroplasty	2017	27593732
Accelerometer-Based Navigation Is as Accurate as Optical Computer Navigation in Restoring the Joint Line and Mechanical Axis After Total Knee Arthroplasty: A Prospective Matched Study.	Goh GS, Liow MH, Lim WS, Tay DK, Yeo SJ, Tan MH.	J Arthroplasty	2016	26220102
Patient's personality predicts recovery after total knee arthroplasty: a retrospective study.	Gong L, Dong JY.	J Orthop Sci	2014	24379015
Patients with Rheumatoid Arthritis have Similar Excellent Outcomes after Total Knee Replacement Compared with Patients with Osteoarthritis.	Goodman SM, Johnson B, Zhang M, Huang WT, Zhu R, Figgie M, Alexiades M, Mandl LA.	J Rheumatol	2016	26628605
Collateral soft tissue release in primary total knee replacement.	Goudie S, Deep K.	Comput Aided Surg	2014	24720493
Flexion contracture following primary total knee arthroplasty: risk factors and outcomes.	Goudie ST, Deakin AH, Ahmad A, Maheshwari R, Picard F.	Orthopedics	2011	22146201

Quality of life outcomes in revision versus primary total knee arthroplasty.	Greidanus NV, Peterson RC, Masri BA, Garbuz DS.	J Arthroplasty	2011	20541360
Increased satisfaction after total knee replacement using sensor-guided technology.	Gustke KA, Golladay GJ, Roche MW, Jerry GJ, Elson LC, Anderson CR.	Bone Joint J	2014	25274917
Increased Range of Motion Is Important for Functional Outcome and Satisfaction After Total Knee Arthroplasty in Asian Patients.	Ha CW, Park YB, Song YS, Kim JH, Park YG.	J Arthroplasty	2016	26777578
Comparative outcomes of total hip and knee arthroplasty: a prospective cohort study.	Hamilton D, Henderson GR, Gaston P, MacDonald D, Howie C, Simpson AH.	Postgrad Med J	2012	22822221
Implant design influences patient outcome after total knee arthroplasty: a prospective double-blind randomised controlled trial.	Hamilton DF, Burnett R, Patton JT, Howie CR, Moran M, Simpson AH, Gaston P.	Bone Joint J	2015	25568415
Discordance between patient and surgeon satisfaction after total joint arthroplasty.	Harris IA, Harris AM, Naylor JM, Adie S, Mittal R, Dao AT.	J Arthroplasty	2013	23462496
Computer navigation vs conventional total knee arthroplasty: five-year functional results of a prospective randomized trial.	Harvie P, Sloan K, Beaver RJ.	J Arthroplasty	2012	21958937
Timing of arthroplasty, what is the influence of nocturnal pain and pain at rest on the outcome?	Haverkamp D, Brokelman RB, van Loon CJ, van Kampen A.	Knee Surg Sports Traumatol Arthrosc	2013	22660972
Lateral subvastus approach with osteotomy of the tibial tubercle for total knee replacement: a two-year prospective, randomised, blinded controlled trial.	Hay GC, Kampshoff J, Kuster MS.	J Bone Joint Surg Br	2010	20513886
Total knee arthroplasties performed with a mini-incision or a standard incision. Similar results at six months follow-up.	Hernandez-Vaquero D, Noriega-Fernandez A, Suarez-Vazquez A.	BMC Musculoskelet Disord	2010	20137094

No differences in functional results and quality of life after single-radius or multiradius TKA.	Hinarejos P, Puig-Verdie L, Leal J, Pelfort X, Torres-Claramunt R, Sanchez-Soler J, Monllau JC.	Knee Surg Sports Traumatol Arthrosc	2016	26658566
Anterolateral approach with tibial tubercle osteotomy versus standard medial approach for primary total knee arthroplasty: does it matter?	Hirschmann MT, Hoffmann M, Krause R, Jenabzadeh RA, Arnold MP, Friederich NF.	BMC Musculoskelet Disord	2010	20649966
Return to Sports Activity following UKA and TKA.	Ho JC, Stitzlein RN, Green CJ, Stoner T, Froimson MI.	J Knee Surg	2016	26166426
An 8- to 10-year review of the Rotaglide total knee replacement.	Horwitz MD, Awan S, Chatoo MB, Stott DJ, Powles DP.	Int Orthop	2009	18084758
Persistent pain after knee replacement: do factors associated with pain vary with degree of patient dissatisfaction?	Howells N, Murray J, Wylde V, Dieppe P, Blom A.	Osteoarthritis Cartilage	2016	27521157
Five-year comparison of oxidized zirconium and cobalt-chromium femoral components in total knee arthroplasty: a randomized controlled trial.	Hui C, Salmon L, Maeno S, Roe J, Walsh W, Pinczewski L.	J Bone Joint Surg Am	2011	21471415
Prosthetic alignment after total knee replacement is not associated with dissatisfaction or change in Oxford Knee Score: A multivariable regression analysis.	Huijbregts HJ, Khan RJ, Fick DP, Jarrett OM, Haebich S.	Knee	2016	26826945
Fast-track surgery for bilateral total knee replacement.	Husted H, Troelsen A, Otte KS, Kristensen BB, Holm G, Kehlet H.	J Bone Joint Surg Br	2011	21357957
Comparison of patellar retention versus resurfacing in LCS mobile-bearing total knee arthroplasty.	Hwang BH, Yang IH, Han CD.	Knee Surg Sports Traumatol Arthrosc	2012	21720892
The impact of obesity on the mid-term outcome of cementless total knee replacement.	Jackson MP, Sexton SA, Walter WL, Walter WK, Zicat BA.	J Bone Joint Surg Br	2009	19651831
Greater Medial Compartment Forces During Total Knee Arthroplasty Associated With Improved Patient Satisfaction and Ability to Navigate Stairs.	Jacobs CA, Christensen CP, Karthikeyan T.	J Arthroplasty	2016	27067762

An Intact Anterior Cruciate Ligament at the Time of Posterior Cruciate Ligament-Retaining Total Knee Arthroplasty Was Associated With Reduced Patient Satisfaction and Inferior Pain and Stair Function.	Jacobs CA, Christensen CP, Karthikeyan T.	J Arthroplasty	2016	26860965
Subchondral Bone Marrow Edema Had Greater Effect on Postoperative Pain After Medial Unicompartmental Knee Arthroplasty Than Total Knee Arthroplasty.	Jacobs CA, Christensen CP, Karthikeyan T.	J Arthroplasty	2016	26474951
Patients with Pain 60 to 120 Days after Total Knee Arthroplasty More Likely to be Dissatisfied at Mid-Term Follow-Up.	Jacobs CA, Christensen CP, Karthikeyan T.	J Arthroplasty	2015	26072301
Chronic Non-Orthopedic Conditions More Common in Patients with Less Severe Degenerative Changes That Have Elected to Undergo Total Knee Arthroplasty.	Jacobs CA, Christensen CP, Karthikeyan T.	J Arthroplasty	2015	25702593
Patient and intraoperative factors influencing satisfaction two to five years after primary total knee arthroplasty.	Jacobs CA, Christensen CP, Karthikeyan T.	J Arthroplasty	2014	24768544
Factors influencing patient satisfaction two to five years after primary total knee arthroplasty.	Jacobs CA, Christensen CP.	J Arthroplasty	2014	24534535
Long-Term Survivorship and Clinical Outcomes Following Total Knee Arthroplasty.	Jauregui JJ, Cherian JJ, Pierce TP, Beaver WB, Issa K, Mont MA.	J Arthroplasty	2015	26100473
Knee arthroplasty with a medial rotating total knee replacement. Midterm clinical findings: a district general experience of 38 cases.	Jonas SC, Argyropoulos M, Al-Hadithy N, Korycki M, Lotz B, Deo SD, Satish V.	Knee	2015	25533210

Interpretation of patient-reported outcomes for hip and knee replacement surgery: identification of thresholds associated with satisfaction with surgery.	Judge A, Arden NK, Kiran A, Price A, Javaid MK, Beard D, Murray D, Field RE.	J Bone Joint Surg Br	2012	22371552
Assessing patients for joint replacement: can pre-operative Oxford hip and knee scores be used to predict patient satisfaction following joint replacement surgery and to guide patient selection?	Judge A, Arden NK, Price A, Glyn-Jones S, Beard D, Carr AJ, Dawson J, Fitzpatrick R, Field RE.	J Bone Joint Surg Br	2011	22161930
Internal rotation of femoral component affects functional activities after TKA--survey with the 2011 Knee Society Score.	Kawahara S, Okazaki K, Matsuda S, Nakahara H, Okamoto S, Iwamoto Y.	J Arthroplasty	2014	24388616
Intermediate-Term Comparison of Posterior Cruciate-Retaining Versus Posterior-Stabilized Total Knee Arthroplasty Using the New Knee Scoring System.	Kawakami Y, Matsumoto T, Takayama K, Ishida K, Nakano N, Matsushita T, Kuroda Y, Patel K, Kuroda R, Kurosaka M.	Orthopedics	2015	26652335
The Cardiff Arthroplasty Satisfaction Index (CASI). A simple tool for the assessment of outcome following knee arthroplasty.	Kempshall PJ, Hickey BA, Metcalfe AJ.	J Arthroplasty	2013	23142443
Socio-economic position has no effect on improvement in health-related quality of life and patient satisfaction in total hip and knee replacement: a cohort study.	Keurentjes JC, Blane D, Bartley M, Keurentjes JJ, Fiocco M, Nelissen RG.	PLoS One	2013	23520456
Patients with severe radiographic osteoarthritis have a better prognosis in physical functioning after hip and knee replacement: a cohort-study.	Keurentjes JC, Fiocco M, So-Osman C, Onstenk R, Koopman-Van Gemert AW, Pöll RG, Kroon HM, Vliet Vlieland TP, Nelissen RG.	PLoS One	2013	23573200
10-Year patient satisfaction compared between computer-assisted navigation and conventional techniques in minimally invasive surgery total knee arthroplasty.	Khuangsirikul S, Lekkreusuwan K, Chotanaphuti T.	Comput Assist Surg (Abingdon)	2016	27973967

Differences in Patient-Reported Outcomes Between Unicompartmental and Total Knee Arthroplasties: A Propensity Score-Matched Analysis.	Kim MS, Koh IJ, Choi YJ, Lee JY, In Y.	J Arthroplasty	2016	27979407
Comparison of patellar resurfacing versus preservation in high flexion total knee arthroplasty.	Kim SH, Lee S, Ro du H, Cho Y, Lee YM, Chung KY, Lee MC.	Knee Surg Sports Traumatol Arthrosc	2015	25223965
The effect of leg length discrepancy on clinical outcome after TKA and identification of possible risk factors.	Kim SH, Rhee SM, Lim JW, Lee HJ.	Knee Surg Sports Traumatol Arthrosc	2016	26597818
Patients still wish for key improvements after total knee arthroplasty.	Kim SJ, Bamne A, Song YD, Kang YG, Kim TK.	Knee Surg Relat Res	2015	25750891
Causes and predictors of patient's dissatisfaction after uncomplicated total knee arthroplasty.	Kim TK, Chang CB, Kang YG, Kim SJ, Seong SC.	J Arthroplasty	2009	18534422
Improved early clinical outcomes of RP/PS mobile-bearing total knee arthroplasties.	Kim TK, Cho HJ, Kang YG, Kim SJ, Chang CB.	Clin Orthop Relat Res	2009	19296186
Functional disabilities and satisfaction after total knee arthroplasty in female Asian patients.	Kim TK, Kwon SK, Kang YG, Chang CB, Seong SC.	J Arthroplasty	2010	19251391
Comparison of a standard and a gender-specific posterior cruciate-substituting high-flexion knee prosthesis: a prospective, randomized, short-term outcome study.	Kim YH, Choi Y, Kim JS.	J Bone Joint Surg Am	2010	20720134
Comparison of standard and gender-specific posterior-cruciate-retaining high-flexion total knee replacements: a prospective, randomised study.	Kim YH, Choi Y, Kim JS.	J Bone Joint Surg Br	2010	20435999
Range of motion of standard and high-flexion posterior cruciate-retaining total knee prostheses a prospective randomized study.	Kim YH, Choi Y, Kim JS.	J Bone Joint Surg Am	2009	19651944

Comparison of the Genesis II total knee replacement with oxidised zirconium and cobalt-chromium femoral components in the same patients: a prospective, double-blind, randomised controlled study.	Kim YH, Park JW, Kim JS.	J Bone Joint Surg Br	2012	22933494
Cementless and cemented total knee arthroplasty in patients younger than fifty five years. Which is better?	Kim YH, Park JW, Lim HM, Park ES.	Int Orthop	2014	24420155
Comparison of highly cross-linked and conventional polyethylene in posterior cruciate-substituting total knee arthroplasty in the same patients.	Kim YH, Park JW.	J Bone Joint Surg Am	2014	25378508
Early outcome of TKA with a medial pivot fixed-bearing prosthesis is worse than with a PFC mobile-bearing prosthesis.	Kim YH, Yoon SH, Kim JS.	Clin Orthop Relat Res	2009	18465188
Total knee arthroplasty in younger patients evaluated by alternative outcome measures.	Klit J, Jacobsen S, Rosenlund S, Sonne-Holm S, Troelsen A.	J Arthroplasty	2014	24269097
Patient-Specific CT-Based Instrumentation versus Conventional Instrumentation in Total Knee Arthroplasty: A Prospective Randomized Controlled Study on Clinical Outcomes and In-Hospital Data.	Kotela A, Lorkowski J, Kucharzewski M, Wilk-Frańczuk M, Śliwiński Z, Frańczuk B, Łęgosz P, Kotela I.	Biomed Res Int	2015	26301241
Primary Total Knee Arthroplasty in Morbidly Obese Patients: a 5- to 14-year follow-up study.	Krushell RJ, Fingeroth RJ.	J Arthroplasty	2007	17823021
Noise Generation With Good Range of Motion but Without Femorotibial Instability Has Small Effect on Patient Satisfaction After Total Knee Arthroplasty.	Kuriyama S, Ishikawa M, Nakamura S, Furu M, Ito H, Matsuda S.	J Arthroplasty	2017	27546474
Subjective evaluation before and after total knee arthroplasty using the 2011 Knee Society Score.	Kuroda Y, Matsumoto T, Takayama K, Ishida K, Kuroda R, Kurosaka M.	Knee	2016	27802922

Interpretations of the clinical outcomes of the nonresponders to mail surveys in patients after total knee arthroplasty.	Kwon SK, Kang YG, Chang CB, Sung SC, Kim TK.	J Arthroplasty	2010	19106032
Correlations between commonly used clinical outcome scales and patient satisfaction after total knee arthroplasty.	Kwon SK, Kang YG, Kim SJ, Chang CB, Seong SC, Kim TK.	J Arthroplasty	2010	20097036
Cementless total knee replacement fixation: a contemporary durable solution--affirms.	Kwong LM, Nielsen ES, Ruiz DR, Hsu AH, Dines MD, Mellano CM.	Bone Joint J	2014	25381416
Factors that shape the patient's hospital experience and satisfaction with lower limb arthroplasty: an exploratory thematic analysis.	Lane JV, Hamilton DF, MacDonald DJ, Ellis C, Howie CR.	BMJ Open	2016	27217282
The efficacy of patellar decompression for improving anterior knee pain following total knee arthroplasty without patellar resurfacing.	Lee GW, Lee SM, Jang SJ, Son JH.	Arch Orthop Trauma Surg	2013	23435648
Clinical outcome using a ligament referencing technique in CAS versus conventional technique.	Lehnen K, Giesinger K, Warschkow R, Porter M, Koch E, Kuster MS.	Knee Surg Sports Traumatol Arthrosc	2011	20852843
Comparison of clinical outcomes between patellar resurfacing and nonresurfacing in total knee arthroplasty: retrospective study of 130 cases.	Li B, Bai L, Fu Y, Wang G, He M, Wang J.	J Int Med Res	2012	23206460
Patient-reported outcomes after total and unicompartmental knee arthroplasty: a study of 14,076 matched patients from the National Joint Registry for England and Wales.	Liddle AD, Pandit H, Judge A, Murray DW.	Bone Joint J	2015	26033059

Are patients more satisfied and have better functional outcome after bilateral total knee arthroplasty as compared to total hip arthroplasty and unilateral total knee arthroplasty surgery? A two-year follow-up study.	Lim JB, Chou AC, Chong HC, Lo NN, Chia SL, Tay KJ, Yeo SJ.	Acta Orthop Belg	2015	26790791
History of previous knee surgery does not affect the clinical outcomes of primary total knee arthroplasty in an Asian population.	Lim JB, Loh B, Chong HC, Tan AH.	Ann Transl Med	2016	27668223
Functional ability, mobility, and pain before and after knee replacement in patients aged 75 and older: a cross-sectional study.	Limnell K, JÃ¤msen E, Huhtala H, JÃ¤ntti P, Puolakka T, JylhÃ¤ M.	Aging Clin Exp Res	2012	23147559
Computer-assisted stereotaxic navigation improves the accuracy of mechanical alignment and component positioning in total knee arthroplasty.	Liow MH, Goh GS, Pang HN, Tay DK, Lo NN, Yeo SJ.	Arch Orthop Trauma Surg	2016	27349233
Robotic-assisted total knee arthroplasty may lead to improvement in quality-of-life measures: a 2-year follow-up of a prospective randomized trial.	Liow MH, Goh GS, Wong MK, Chin PL, Tay DK, Yeo SJ.	Knee Surg Sports Traumatol Arthrosc	2016	27017214
Negative impact of waiting time for primary total knee arthroplasty on satisfaction and patient-reported outcome.	Lizaur-Utrilla A, Martinez-Mendez D, Miralles-MuÃ±oz FA, Marco-Gomez L, Lopez-Prats FA.	Int Orthop	2016	27121844
Greater satisfaction in older patients with a mobile-bearing compared with fixed-bearing total knee arthroplasty.	Lizaur-Utrilla A, Sanz-Reig J, Trigueros-Rentero MA.	J Arthroplasty	2012	21752586
Pain and function in patients after primary unicompartmental and total knee arthroplasty.	Lygre SH, Espehaug B, Havelin LI, Furnes O, Vollset SE.	J Bone Joint Surg Am	2010	21159989

Does patella resurfacing really matter? Pain and function in 972 patients after primary total knee arthroplasty.	Lygre SH, Espehaug B, Havelin LI, Vollset SE, Furnes O.	Acta Orthop	2010	20158405
Functional Outcomes of a New Mobile-Bearing Ultra-Congruent TKA System: Comparison With the Posterior Stabilized System.	Machhindra MV, Kang JY, Kang YG, Chowdhry M, Kim TK.	J Arthroplasty	2015	26187388
The self-administered patient satisfaction scale for primary hip and knee arthroplasty.	Mahomed N, Gandhi R, Daltroy L, Katz JN.	Arthritis	2011	22046521
The role of patient expectations in predicting outcome after total knee arthroplasty.	Mannion AF, KÃ¤mpfen S, Munzinger U, Kramers-de Quervain I.	Arthritis Res Ther	2009	19772556
Predictors of Satisfaction Following Total Knee Arthroplasty.	Maratt JD, Lee YY, Lyman S, Westrich GH.	J Arthroplasty	2015	25680451
Postoperative alignment and ROM affect patient satisfaction after TKA.	Matsuda S, Kawahara S, Okazaki K, Tashiro Y, Iwamoto Y.	Clin Orthop Relat Res	2013	22903282
A Cohort Study Predicts Better Functional Outcomes and Equivalent Patient Satisfaction Following UKR Compared with TKR.	Matthews DJ, Hossain FS, Patel S, Haddad FS.	HSS J	2013	24426840
Patient-Reported Allergies Are Associated With Poorer Patient Satisfaction and Outcomes After Lower Extremity Arthroplasty: A Retrospective Cohort Study.	McLawhorn AS, Bjerke-Kroll BT, Blevins JL, Sculco PK, Lee YY, Jerabek SA.	J Arthroplasty	2015	25702595
Safety and efficacy of a rotating-platform, high-flexion knee design three- to five-year follow-up.	Meftah M, Ranawat AS, Ranawat CS.	J Arthroplasty	2012	21621961
Long-term results of total knee arthroplasty in young and active patients with posterior stabilized design.	Meftah M, White PB, Ranawat AS, Ranawat CS.	Knee	2016	26833096
Surgeon's expectations do not predict the outcome of a total knee arthroplasty.	Meijerink HJ, Brokelman RB, van Loon CJ, van Kampen A, de Waal Malefijt MC.	Arch Orthop Trauma Surg	2009	19099309

Similar TKA designs with differences in clinical outcome: a randomized, controlled trial of 77 knees with a mean follow-up of 6 years.	Meijerink HJ, Verdonschot N, van Loon CJ, Hannink G, de Waalmalefijt MC.	Acta Orthop	2011	22066559
Can Intraoperative Sensors Determine the "Target" Ligament Balance? Early Outcomes in Total Knee Arthroplasty.	Meneghini RM, Ziemba-Davis MM, Lovro LR, Ireland PH, Damer BM.	J Arthroplasty	2016	27155997
Factors predicting patient satisfaction 2 years after total knee arthroplasty for osteoarthritis.	Merle-Vincent F, Couris CM, Schott AM, Conrozier T, Piperno M, Mathieu P, Vignon E; Osteoarthritis Section of the French Society for Rheumatology.	Joint Bone Spine	2011	21196128
Knee arthroplasties have similar results in high- and low-activity patients.	Mont MA, Marker DR, Seyler TM, Gordon N, Hungerford DS, Jones LC.	Clin Orthop Relat Res	2007	17310928
High-impact sports after total knee arthroplasty.	Mont MA, Marker DR, Seyler TM, Jones LC, Kolisek FR, Hungerford DS.	J Arthroplasty	2008	18722307
The effect of the native kinematics of the knee on the outcome following total knee arthroplasty.	Mooney LT, Smith A, Sloan K, Clark GW.	Bone Joint J	2016	27803222
Does flexion of the femoral implant in total knee arthroplasty increase knee flexion: a randomised controlled trial.	Murphy M, Journeaux S, Hides J, Russell T.	Knee	2014	23183371
Clinical improvement and satisfaction after total joint replacement: a prospective 12-month evaluation on the patients' perspective.	Naal FD, Impellizzeri FM, Lenze U, Wellauer V, von Eisenhart-Rothe R, Leunig M.	Qual Life Res	2015	26068733
Correlations between patient satisfaction and ability to perform daily activities after total knee arthroplasty: why aren't patients satisfied?	Nakahara H, Okazaki K, Mizu-Uchi H, Hamai S, Tashiro Y, Matsuda S, Iwamoto Y.	J Orthop Sci	2015	25366699
Long-term subjective outcomes of computer-assisted total knee arthroplasty.	Nakano N, Matsumoto T, Ishida K, Tsumura N, Kuroda R, Kurosaka M.	Int Orthop	2013	23807729

Residual Symptoms and Function After Unicompartmental and Total Knee Arthroplasty: Comparable to Normative Controls?	Nam D, Berend ME, Nunley RM, Della Valle CJ, Berend KR, Lombardi AV, Barrack RL.	J Arthroplasty	2016	27067170
Patient-Reported Metal Allergy: A Risk Factor for Poor Outcomes After Total Joint Arthroplasty?	Nam D, Li K, Riegler V, Barrack RL.	J Arthroplasty	2016	26965589
Patient dissatisfaction following total knee replacement: a growing concern?	Nam D, Nunley RM, Barrack RL.	Bone Joint J	2014	25381418
The impact of custom cutting guides on patient satisfaction and residual symptoms following total knee arthroplasty.	Nam D, Nunley RM, Berend KR, Lombardi AV, Barrack RL.	Knee	2016	26746042
Total knee replacement and health-related quality of life: factors influencing long-term outcomes.	Núñez M, Lozano L, Núñez E, Segur JM, Sastre S, Maculé F, Ortega R, Suso S.	Arthritis Rheum	2009	19644900
Is extreme flexion of the knee after total knee replacement a prerequisite for patient satisfaction?	Narayan K, Thomas G, Kumar R.	Int Orthop	2009	18509639
Patients' Expectations Impact Their Satisfaction following Total Hip or Knee Arthroplasty.	Neuprez A, Delcour JP, Fatemi F, Gillet P, Crielaard JM, BruyÃ¨re O, Reginster JY.	PLoS One	2016	27977711
Knee arthroplasty: are patients' expectations fulfilled? A prospective study of pain and function in 102 patients with 5-year follow-up.	Nilsdotter AK, Toksvig-Larsen S, Roos EM.	Acta Orthop	2009	19234886
Intraoperative medial pivot affects deep knee flexion angle and patient-reported outcomes after total knee arthroplasty.	Nishio Y, Onodera T, Kasahara Y, Takahashi D, Iwasaki N, Majima T.	J Arthroplasty	2014	23958237
New total knee arthroplasty designs: do young patients notice?	Nunley RM, Nam D, Berend KR, Lombardi AV, Dennis DA, Della Valle CJ, Barrack RL.	Clin Orthop Relat Res	2015	24903823
Influence of depression on total knee arthroplasty outcomes.	Pérez-Prieto D, Gil-González S, Pelfort X, Leal-Blanquet J, Puig-Verdié L, Hinarejos P.	J Arthroplasty	2014	23702267

Simultaneous cemented and cementless total knee replacement in the same patients: a prospective comparison of long-term outcomes using an identical design of NexGen prosthesis.	Park JW, Kim YH.	J Bone Joint Surg Br	2011	22058298
High level of residual symptoms in young patients after total knee arthroplasty.	Parvizi J, Nunley RM, Berend KR, Lombardi AV Jr, Ruh EL, Clohisy JC, Hamilton WG, Della Valle CJ, Barrack RL.	Clin Orthop Relat Res	2014	24061845
Inferior outcomes of total knee replacement in early radiological stages of osteoarthritis.	Peck CN, Childs J, McLauchlan GJ.	Knee	2014	25205527
Patella denervation in primary total knee arthroplasty - a randomized controlled trial with 2 years of follow-up.	Pulavarti RS, Raut VV, McLauchlan GJ.	J Arthroplasty	2014	24291230
Clinical and Radiographic Results of Attune and PFC Sigma Knee Designs at 2-Year Follow-Up: A Prospective Matched-Pair Analysis.	Ranawat CS, White PB, West S, Ranawat AS.	J Arthroplasty	2017	27600300
Association between Neuropathic Pain and Reported Disability after Total Knee Arthroplasty.	Razmjou H, Boljanovic D, Wright S, Murnaghan J, Holtby R.	Physiother Can	2015	27504030
Selective patellar resurfacing in total knee arthroplasty: a prospective, randomized, double-blind study.	Roberts DW, Hayes TD, Tate CT, Lesko JP.	J Arthroplasty	2015	25316378
A 15-year follow-up study of 4606 primary total knee replacements.	Roberts VI, Esler CN, Harper WM.	J Bone Joint Surg Br	2007	17998180
Bicruciate substituting design does not improve maximal flexion in total knee arthroplasty: a randomized controlled trial.	Schimmel JJ, Defoort KC, Heesterbeek PJ, Wymenga AB, Jacobs WC, van Hellemondt GG.	J Bone Joint Surg Am	2014	24875033
Pre-operative arthritis severity as a predictor for total knee arthroplasty patients' satisfaction.	Schnurr C, Jarrous M, Güdden I, Eysel P, König DP.	Int Orthop	2013	23525526

Anteroposterior stability after posterior cruciate-retaining total knee arthroplasty.	Schuster AJ, von Roll AL, Pfluger D, Wyss T.	Knee Surg Sports Traumatol Arthrosc	2011	21234540
Patient expectations of arthroplasty of the hip and knee.	Scott CE, Bugler KE, Clement ND, MacDonald D, Howie CR, Biant LC.	J Bone Joint Surg Br	2012	22733956
Five-year survivorship and patient-reported outcome of the Triathlon single-radius total knee arthroplasty.	Scott CE, Clement ND, MacDonald DJ, Hamilton DF, Gaston P, Howie CR, Burnett R.	Knee Surg Sports Traumatol Arthrosc	2015	24623184
Predicting dissatisfaction following total knee replacement: a prospective study of 1217 patients.	Scott CE, Howie CR, MacDonald D, Biant LC.	J Bone Joint Surg Br	2010	20798443
Staged bilateral total knee replacement: changes in expectations and outcomes between the first and second operations.	Scott CE, Murray RC, MacDonald DJ, Biant LC.	Bone Joint J	2014	24891574
Predicting dissatisfaction following total knee arthroplasty in patients under 55 years of age.	Scott CE, Oliver WM, MacDonald D, Wade FA, Moran M, Breusch SJ.	Bone Joint J	2016	27909124
Does patellofemoral congruence following total knee arthroplasty correlate with pain or function? Intraoperative arthroscopic assessment of 30 cases.	Senioris A, Saffarini M, Rahali S, Malekpour L, Dujardin F, Courage O.	Ann Transl Med	2016	27570773
Is Total Knee Arthroplasty a Viable Treatment Option in Octogenarians with Advanced Osteoarthritis?	Seo JG, Moon YW, Cho BC, Kim SC, Ko YH, Jang SP, Lee BH.	Knee Surg Relat Res	2015	26675818
Noise, numbness, and kneeling difficulties after total knee arthroplasty: is the outcome affected?	Sharkey PF, Miller AJ.	J Arthroplasty	2011	21256704
Outcome of Total Knee Replacement via Two Approaches in Indian Scenario.	Shukla R, Mahajan P, Singh M, Jain RK, Kumar R.	J Knee Surg	2016	27213285
Navigation-assisted versus conventional total knee replacement: no difference in patient-reported outcome measures (PROMs) at 1 and 2 years.	Singisetti K, Muthumayandi K, Abual-Rub Z, Weir D.	Arch Orthop Trauma Surg	2015	26303281

High patient satisfaction in 445 patients who underwent fast-track hip or knee replacement.	Specht K, Kjaersgaard-Andersen P, Kehlet H, Wedderkopp N, Pedersen BD.	Acta Orthop	2015	26109124
Patelloplasty versus traditional total knee arthroplasty for osteoarthritis.	Sun YQ, Yang B, Tong SL, Sun J, Zhu YC.	Orthopedics	2012	22385444
Patient satisfaction after total knee arthroplasty: an Asian perspective.	Thambiah MD, Nathan S, Seow BZ, Liang S, Lingaraj K.	Singapore Med J	2015	26034317
Do we need a gender-specific total knee replacement? A randomised controlled trial comparing a high-flex and a gender-specific posterior design.	Thomsen MG, Husted H, Bencke J, Curtis D, Holm G, Troelsen A.	J Bone Joint Surg Br	2012	22628593
Do patients care about higher flexion in total knee arthroplasty? A randomized, controlled, double-blinded trial.	Thomsen MG, Husted H, Otte KS, Holm G, Troelsen A.	BMC Musculoskelet Disord	2013	23565578
How gait and clinical outcomes contribute to patients' satisfaction three months following a total knee arthroplasty.	Turcot K, Sagawa Y Jr, Fritschy D, Hoffmeyer P, SuvÃ D, Armand S.	J Arthroplasty	2013	23528552
Effect of medial-lateral malpositioning of the femoral component in total knee arthroplasty on anterior knee pain at greater than 8 years of follow-up.	van de Groes SA, Koëter S, de Waal Malefijt M, Verdonschot N.	Knee	2014	25199450
All-polyethylene tibial components in octogenarians: survivorship, performance, and cost.	van der Ven A, Scott RD, Barnes CL.	Am J Orthop (Belle Mead NJ)	2014	24490181
Patella position is not a determinant for anterior knee pain 10 years after balanced gap total knee arthroplasty.	van Houten AH, Heesterbeek PJ, Wymenga AB.	Knee Surg Sports Traumatol Arthrosc	2016	26704792
A New Prediction Model for Patient Satisfaction After Total Knee Arthroplasty.	Van Onsem S, Van Der Straeten C, Arnout N, Deprez P, Van Damme G, Victor J.	J Arthroplasty	2016	27506723
Patient satisfaction at 2 months following total knee replacement using a second generation medial-pivot system: follow-up of 250 consecutive cases.	Van Overschelde PP, Fitch DA.	Ann Transl Med	2016	27761443

Functional capacity and actual daily activity do not contribute to patient satisfaction after total knee arthroplasty.	Vissers MM, de Groot IB, Reijman M, Bussmann JB, Stam HJ, Verhaar JA.	BMC Musculoskelet Disord	2010	20553584
LCS mobile-bearing total knee replacement. A 10-year's follow-up study.	Vogt JC, Saarbach C.	Orthop Traumatol Surg Res	2009	19328763
Patient satisfaction after primary total and unicompartmental knee arthroplasty: an age-dependent analysis.	Von Keudell A, Sodha S, Collins J, Minas T, Fitz W, Gomoll AH.	Knee	2014	24148793
Functional and radiographic short-term outcome evaluation of the Visionaire system, a patient-matched instrumentation system for total knee arthroplasty.	Vundelinckx BJ, Bruckers L, De Mulder K, De Schepper J, Van Esbroeck G.	J Arthroplasty	2013	23535285
Results of Lateral Retinacular Release Plus Circumpatellar Electrocautery in Total Knee Arthroplasty without Patellar Resurfacing.	Wang Y, Sun JY, Zha GC.	J Knee Surg	2016	27206069
Similar outcome for total knee arthroplasty after previous high tibial osteotomy and for total knee arthroplasty as the first measure.	W-Dahl A, Robertsson O.	Acta Orthop	2016	27339330
Patient-Specific Total Knees Demonstrate a Higher Manipulation Rate Compared to "Off-the-Shelf Implants".	White PB, Ranawat AS.	J Arthroplasty	2016	26318082
Early postoperative predictors of satisfaction following total knee arthroplasty.	Williams DP, O'Brien S, Doran E, Price AJ, Beard DJ, Murray DW, Beverland DE.	Knee	2013	23777807
Patient-reported outcomes after fixed- versus mobile-bearing total knee replacement: a multi-centre randomised controlled trial using the Kinemax total knee replacement.	Wylde V, Learmonth I, Potter A, Bettinson K, Lingard E.	J Bone Joint Surg Br	2008	18757956

Tension controlled ligament balanced total knee arthroplasty: 5-year results of a soft tissue orientated surgical technique.	Wyss T, Schuster AJ, Christen B, Wehrli U.	Arch Orthop Trauma Surg	2008	18075748
High-flex posterior cruciate-retaining vs posterior cruciate-substituting designs in simultaneous bilateral total knee arthroplasty: a prospective, randomized study.	Yagishita K, Muneta T, Ju YJ, Morito T, Yamazaki J, Sekiya I.	J Arthroplasty	2012	21680139
Less anterior knee pain with a routine lateral release in total knee arthroplasty without patellar resurfacing: a prospective, randomized study.	Zha GC, Sun JY, Dong SJ.	Knee Surg Sports Traumatol Arthrosc	2014	24288077
Total knee arthroplasty for severe valgus knee deformity.	Zhou X, Wang M, Liu C, Zhang L, Zhou Y.	Chin Med J (Engl)	2014	24622435
